# The Circulating Proteome—Technological Developments, Current Challenges, and Future Trends

**DOI:** 10.1021/acs.jproteome.4c00586

**Published:** 2024-10-31

**Authors:** Philipp E. Geyer, Daniel Hornburg, Maria Pernemalm, Stefanie M. Hauck, Krishnan K. Palaniappan, Vincent Albrecht, Laura F. Dagley, Robert L. Moritz, Xiaobo Yu, Fredrik Edfors, Yves Vandenbrouck, Johannes B. Mueller-Reif, Zhi Sun, Virginie Brun, Sara Ahadi, Gilbert S. Omenn, Eric W. Deutsch, Jochen M. Schwenk

**Affiliations:** † Department of Proteomics and Signal Transduction, Max Planck Institute of Biochemistry, 82152 Martinsried, Germany; ‡ Seer, Inc., Redwood City, California 94065, United States; § Bruker Scientific, San Jose, California 95134, United States; ∥ Department of Oncology and Pathology/Science for Life Laboratory, Karolinska Institutet, 17177 Stockholm, Sweden; ⊥ Metabolomics and Proteomics Core, Helmholtz Zentrum München GmbH, German Research Center for Environmental Health, 85764 Oberschleissheim, Munich, Germany; # Freenome, South San Francisco, California 94080, United States; ¶ The Walter and Eliza Hall Institute for Medical Research, Parkville, VIC 3052, Australia; □ Department of Medical Biology, University of Melbourne, Parkville, VIC 3052, Australia; ■ Institute for Systems Biology, Seattle, Washington 98109, United States; ○ State Key Laboratory of Medical Proteomics, Beijing Proteome Research Center, National Center for Protein Sciences-Beijing (PHOENIX Center), Beijing Institute of Lifeomics, Beijing 102206, China; ● Science for Life Laboratory, Department of Protein Science, KTH Royal Institute of Technology, 17121 Solna, Sweden; △ CEA, Fundamental Research Division, 91191 Gif-sur-Yvette, France; ▲ Université Grenoble Alpes, CEA, Leti, Clinatec, Inserm UA13 BGE, CNRS FR2048, Grenoble, France; ▽ Alkahest, Inc., Suite D San Carlos, California 94070, United States; ▼ Departments of Computational Medicine & Bioinformatics, Internal Medicine, Human Genetics and Environmental Health, University of Michigan, Ann Arbor, Michigan 48109-2218, United States

**Keywords:** biomarker discovery, blood, plasma, serum, PTM, extracellular vesicle, microsampling, affinity, mass spectrometry, PeptideAtlas

## Abstract

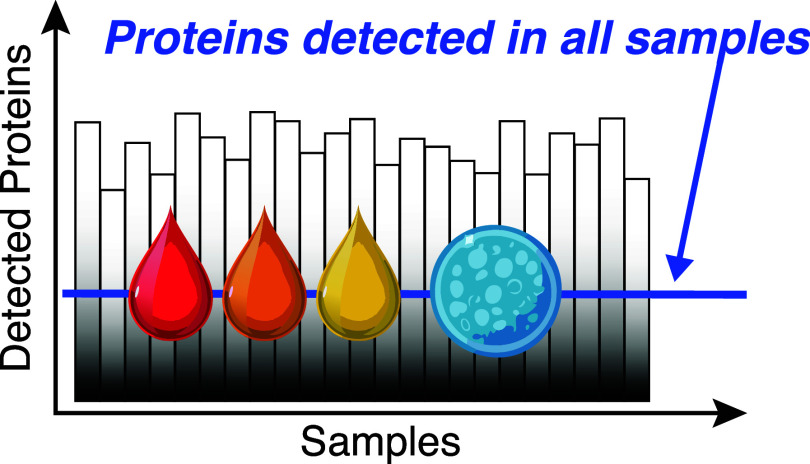

Recent improvements in proteomics technologies have fundamentally altered our capacities to characterize human biology. There is an ever-growing interest in using these novel methods for studying the circulating proteome, as blood offers an accessible window into human health. However, every methodological innovation and analytical progress calls for reassessing our existing approaches and routines to ensure that the new data will add value to the greater biomedical research community and avoid previous errors. As representatives of HUPO’s Human Plasma Proteome Project (HPPP), we present our 2024 survey of the current progress in our community, including the latest build of the Human Plasma Proteome PeptideAtlas that now comprises 4608 proteins detected in 113 data sets. We then discuss the updates of established proteomics methods, emerging technologies, and investigations of proteoforms, protein networks, extracellualr vesicles, circulating antibodies and microsamples. Finally, we provide a prospective view of using the current and emerging proteomics tools in studies of circulating proteins.

## Introduction

The circulating proteome, consisting of the ensemble of all proteins possibly present in blood plasma or serum, holds immense potential to understand biological and pathological effects, including the promise of finding novel biomarkers that can be utilized for health and disease assessment. However, the extraordinary complexity of the circulating proteome, including various types of biomolecules and the extensive dynamic range of protein abundances spanning over 12 orders of magnitude, has posed substantial analytical challenges for decades.^1−4^ As the community strives forward, we have seen remarkable advancements in affinity-based and mass spectrometry (MS)-based technologies for increasingly higher proteome coverage and throughput. This has been methodology driven by the need to address complex research questions, such as investigating the flow of information and relationships between the genome and proteome and the inherent biological variation among individuals.

As proteomic analyses of blood proteins are becoming more accessible and easier to integrate into non-proteomic studies, the community can now begin to address the remaining hurdles and opportunities. Several studies have shown that the intraindividual circulating proteome stability over time is high, but interindividual variability is significant.^5,6^ These observations emphasize the importance of analyzing larger sample numbers, whether from cross-sectional studies with larger donor cohorts or through longitudinal blood collections.^7^ Consequently, this underscores the necessity of solidifying capabilities for processing extensive sample sets with high precision and utilizing data from complementary analytical concepts. Methods like Mendelian randomization have identified druggable proteins with causal relationships to disease.^8^ Study design and various technical and biological factors need to be considered to reduce overall variability, facilitating the discovery of physiologically relevant signatures.

Here, we aim to discuss recent technological developments in the exploration of the circulating proteome and provide a reference database of proteins currently detected through an update of the PeptideAtlas. Finally, we offer a prospective view into using the current and emerging proteomics tools in studies of proteins in plasma and serum samples to highlight current trends in the study of circulating proteins and proteoforms.

## Affinity-Based Platforms to Profile the Circulating Proteome

Affinity-based approaches limit surveying the proteome to a predefined set of proteins. According to Antibodypedia (https://www.antibodypedia.com), nearly five million affinity reagent products are available, covering 95% of the ∼20 000 protein-encoding genes. Over the past few years, two highly multiplexed affinity assay concepts have gained momentum for large-scale investigations.^9^ The Olink proximity extension assays (PEA) exceed 5400 complementary pairs of oligonucleotide-coupled antibodies,^10^ and the SomaLogic slow off-rate aptamer (SOMAmers)^11^ platform now encompasses 11 000 modified aptamers. Both commercially available systems have been used across hundreds of studies, including single projects encompassing tens of thousands of samples.^12−14^ While the aptamer-based assays capture proteins on beads, followed by extensive washing and hybridization of the aptamer to a DNA microarray, Olink’s dual antibody assay relies on the proximal binding of two antibodies to a common target protein epitope in solution, followed by hybridization and PCR amplification of DNA barcodes with a readout by qPCR or NGS.^10,15^ Similar to the latter, the recently introduced NULISA platform of Alamar Biosciences uses the concept of in-solution binding combined with capture steps of the formed immunocomplex (via the oligo and biotin tags) to stringently wash away unbound proteins to improve sensitivity.^16^ All these methods require sample type and assay-specific validation efforts to achieve a selective detection of the intended target with the needed affinity to quantify low amounts. Consequently, in-house pipelines to produce affinity binders (as classical antibodies or alternative scaffolds) have been initiated to enable a more comprehensive exploration of the proteome at scale. The content of these panels has recently grown to several thousand features per sample, enabling users to uncover new biology.^17^ It is worth noting that the frequency of observing all newly added proteins above the limit of detection or quantification in all samples and phenotypes will have to be determined.

A prominent topic in the community has been validating the findings from highly multiplexed affinity assays. There have been concerns about the specificity of the binders in terms of the intended proteins of interest and the availability of the affinity binders used for downstream validation analyses. Even though correlating data from one method with another is regarded as the ideal comparison, growing knowledge about other influential determinants, such as analytical performance or association with genetic variation, has made these investigations more delicate.^18,19^ As shown in a recent study identifying the protein CFHR5 in venous thrombosis, one approach is to follow initial discoveries with an extensive suite of proteomics and functional experiments, including tests for causality.^20^ However, targets can often only be detected on one platform or assay or require alternative concepts to infer their selectivity.^21^ Testing the associations between the sequence variance of the whole genome with the portfolio of protein assay data has provided such opportunities (given that there is an association between the protein-encoding gene and its protein product).^9^ In pursuit of the ground truth, complementary techniques such as mass spectrometry and biological verification should be employed, taking into account the aforementioned factors and potential preanalytical biological influences (see below).

## Large-Scale Comparative Analyses of Affinity-Based Platforms

**Figure 1:**
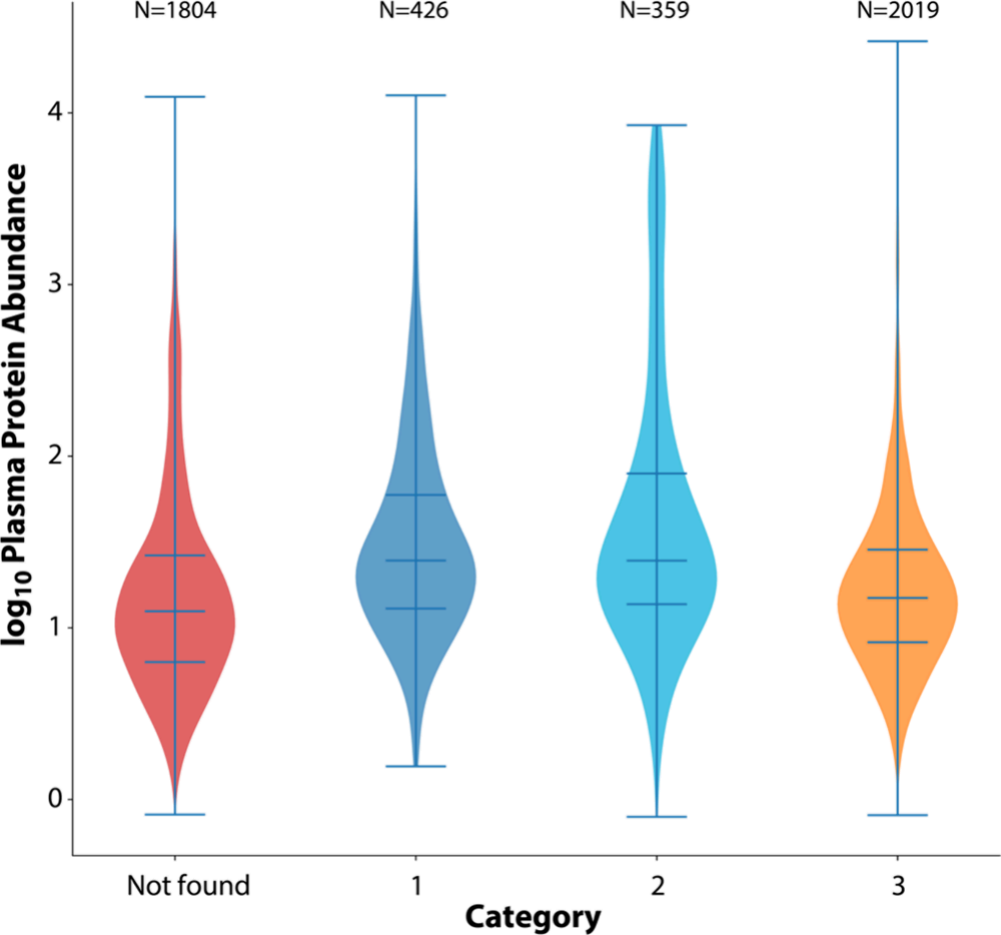
Protein abundance and confidence of detection by affinity assays. The boxplots show the MS-based abundance levels from the 2023 Human Plasma PeptideAtlas build and proteins classified by Eldjarn et al.^22^ into confidence tiers (categories) when comparing data from Olink and SomaLogic assays. Proteins with higher support from cross-platform evidence (tier 1 and 2) have a higher abundance than proteins of lower confidence (tier 3) or those that were not detected by these affinity assays but were part of the 2023 Human Plasma PeptideAtlas build (Not found).

There has been a growing interest in comparing the data originating from large-scale affinity-based analyses.^18,19^ In the most recent and extensive analysis, Eldjarn et al.^22^ invested in the concordance between protein profiles and their genetic associations using the Olink and SomaLogic platforms. They found 576 proteins with high correlation and cis-pQTLs for the intended target (tier 1) and another 515 proteins that had a lower correlation but cis-pQTLs on either platform with a lack in protein level correlation (tier 2). As shown in Figure 1 (74%—426/576—of proteins in tier 1 and 70%—359/515—of tier 2 were listed in the 2023 PeptideAtlas build. Compared to tier 1, proteins either lacking a cis-pQTL (tier 3, Kolmogorov–Smirnov *p* = 4 × 10^–15^) or those being listed in the build of 2023 were generally of lower abundance (*p* = 6 × 10^–24^). Many of the proteins detected at low abundance in blood are of intracellular origin or membrane bound. Other low-abundant and secreted proteins are likely related to inflammatory processes, which may require a specific phenotype to increase protein levels to a detectable range. This suggests that there are about 785 (426 + 359) proteins with supportive evidence from affinity proteomics assays that could be detectable by mass spectrometry. This set of proteins offers a valuable resource for comparing the outcome of investigations conducted on different platforms. It is expected, however, that some findings may still differ due to sensitivity, PTMs, or epitope effects.^9^

## MS-Based Platforms to Profile the Circulating Proteome

In contrast to affinity-based technologies, mass spectrometry analysis does not require a predefined set of targeted proteins. However, global MS-based analysis of the circulating proteome has been hampered by challenges in sensitivity due to the high dynamic range of protein concentrations. Consequently, few studies report over 2000 proteins detected robustly across larger data sets.^23^ However, in 2023, a new mass analyzer, the Astral,^24^ was introduced. Initial studies presented up to today show promising results regarding proteome coverage with over 1000 proteins in neat plasma and even more extensive proteome coverage of >4000 proteins when combined with additional enrichment methods.^25,26^

In addition to the advances in MS instrumentation, automation of sample preparation with liquid-handling platforms has been rolled out to many laboratories, scaling up throughput by parallelizing previously tedious manual work.^27−30^ Automated sample preparation further allowed the implementation of ISO13485 standardization and facilitated proteomics in regulated clinical laboratories.^28^ Analyzing larger sample sets calls for robustness across sample preparation workflows, LC-MS platforms, and scalable computational pipelines to enable reliable identification and quantification of proteins in all samples. This demand has driven the development of novel LC systems and the adaption of analytical flow rates (e.g., high and microflow) and shorter gradients.^31−33^ Combining these with more sensitive mass spectrometers and employing new MS acquisition strategies allowed for quantitative monitoring of over a hundred samples daily.^28^ Especially, data independent acquisition (DIA) has shown its ability to consistently cover high numbers of quantified proteins across samples, leading to the application of this scanning mode in numerous studies. Of note, the robustness and constant high performance of the mass spectrometers themselves have substantially improved in the past few years. Here, especially the Bruker timsTOF HT series has demonstrated its endurance in several large-scale plasma proteomics studies.^34−36^ Besides higher sample throughput, such approaches reduce overall costs per sample, increase the system’s stability, and achieve the desired quantitative accuracy of all detected markers. Larger cohorts not only increase statistical power and are the means against biological variation, but they also allow the application of machine learning to proteomics data.^37,38^ Advances and innovations in instrumentation, sample-processing concepts, and analytical approaches are expected to emerge and be adopted in the coming years.

The unique analytical challenge of handling the wide concentration range of the plasma proteome has been a constant challenge for the field. It has, therefore, been crucial to utilize technologies capable of efficiently sampling across a broad spectrum of protein abundances. These include depleting the most abundant proteins with mainly commercial solutions of antibodies bound to a solid chromatographic phase^39,40^ and investigating extracellular vesicles (see section below). Like sample preparation, these methods are automatable. Regarding the cost of sample throughput, proteome coverage can be increased by fractionation.^41^ More recently, an automated workflow has been introduced to compress the large dynamic range of protein abundances by exploiting the competitive binding of proteins at the nanobiointerface of superparamagnetic functionalized nanoparticles (NPs).^42^ Thus far, it has enabled thousands of circulating proteins to be surveyed within hours, promising scalability to thousands of subjects.^43,44^ Unlike antibody-based depletion strategies, these engineered NPs probe the organism-agnostic physicochemical properties of entire proteomes without preselecting proteins of interest when combined with unbiased LC-MS. This has started a trend, and similar enrichment strategies are entering the field, often driven by commercial vendors. In addition to enrichment and depletion, chemical treatment of serum or plasma has been proposed to enhance the number of quantified proteins. In this approach, proteins are precipitated by perchloric acid, and analysis of the supernatant has revealed up to 1300 proteins per sample. The simplicity of the precipitation allowed early automation and scaling of the analysis to process 3000 samples.^36,45^ In the initial publications, the technical reproducibility of the bead-based enrichment and the precipitation methods seemed promising, and proteins linked to different disease conditions could be identified. A wider adaptation of the methods outside of the developers’ laboratories will show how useful and reliable these methods will be in the community. As is the case for all highly sensitive workflows, an improved sensitivity requires an even more careful investigation of preanalytical variability to ensure confident biological or pathological interpretation. Here, untargeted MS readouts have the advantage that they should be able to detect the contribution of preanalytical variation, such as platelet proteins, and tools can be developed to ensure the quality of the results.^46^

## Future of Targeted MS-Based Proteomics

In addition to collecting all available peptide data by unbiased MS, targeted MS-proteomics offers an approach to quantify preselected peptides consistently. Thus, its applications are tailored closer to clinical applications, which mainly rely on the validation of discovery-driven findings.^47^ Targeted MS can quantify proteins with high precision, sensitivity, specificity, and throughput. The technology is often combined with spike-in of stable isotope-labeled peptide standards (SIS) to obtain absolute quantification. Serial dilution of the standard will also allow for absolute concentrations to be determined. Targeting has typically been limited in sensitivity and the quantification of less than 100 proteins. However, this has changed in recent years, and multiplex SIS panels of peptides or proteins have become increasingly useful due to their ability to measure hundreds of proteins in a single assay.^48,49^ Larger multiplex panels make these concepts increasingly relevant for discovery-focused applications, similar to what current highly multiplexed affinity-based platforms offer with predefined content (panels). The applicability of targeted MS-based assays for disease classification has been demonstrated as a response to the COVID-19 pandemic.^50−52^ Additionally, the development of the novel hybrid high-speed Stellar MS shows the rapid advancements of the technology tailored toward future clinical applications. The Stellar MS demonstrates high reproducibility, sensitivity, and specificity, sufficient for over 1000 plasma proteins, and has been applied to targeted assays for alcohol-related liver disease biomarkers.^53^

The development of user-friendly, pretitrated mixtures of SIS peptides by various vendors has made incorporating them into various workflows easier. The ease of use has increased further thanks to software advancements with intelligent acquisition methods for ion selection and on-the-flight monitoring enabled by methods such as SureQuant,^54^ Pseudo-PRM,^55^ TOMAHAQ (triggered-by-offset, multiplexed, accurate-mass, high-resolution, and absolute quantification),^56^ Scout-MRM,^57^ and MaxQuant.Live.^58^ These strategies increase the mass spectrometer’s ability to concentrate data acquisition time on spiked SIS target peptides, resulting in an even more sensitive detection and a higher number of targets acquired per run.

Commercially available SIS panels often lack sufficient signature peptides per protein, causing downstream quantitative biases when only one peptide is used for the absolute concentration determination. This directly affects assay accuracy when samples must be removed due to missing data or when basing the concentration determination on different peptides.^59,60^ New standards based on full-length proteins or fragments can address this issue and compensate for potential biases introduced during sample preparation.^61^ With growing awareness about how genetic variation can affect protein detection, these issues must be carefully addressed to fully realize the potential of targeted proteomics in diagnostic and clinical settings.

## Community Data

**Figure 2:**
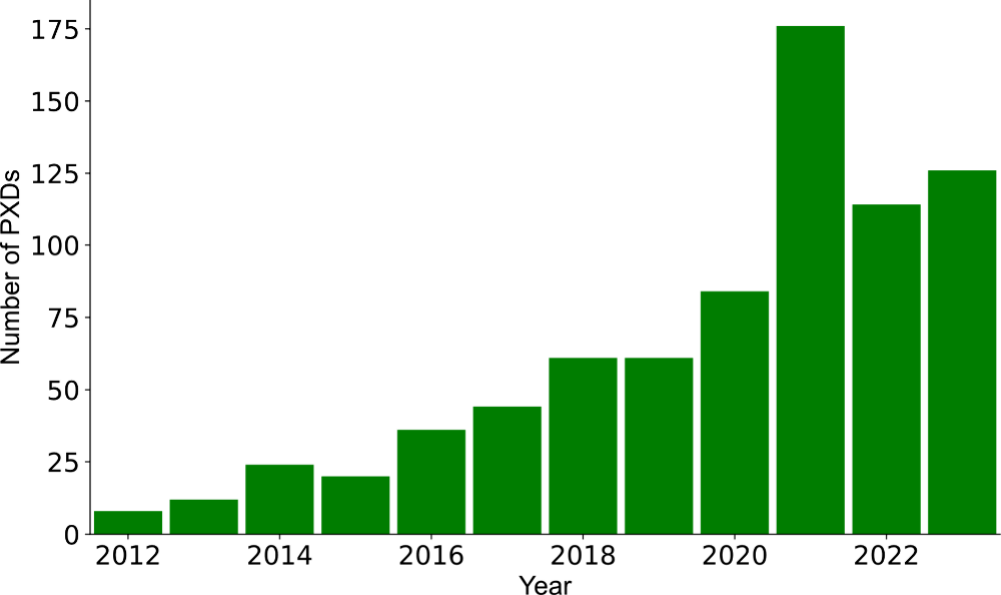
Number of data set submissions to ProteomeXchange over time. The submissions include only MS-based proteomics data sets. The increase in 2021 is explained by the release of the Blood Proteoform Atlas data sets.^64^

Lastly, we briefly touch upon the tools that have enabled the community to follow the progress via the generated data. The general culture of MS data sharing has been well established in the proteomics community for several years,^62^ primarily driven by the efforts of the ProteomeXchange Consortium of proteomics data repositories. ProteomeXchange submissions now number approximately 600 data sets per month.^63^ As shown in Figure 2, there has been an increasing number of submissions from studies using plasma samples. This data sharing has accelerated the development of open-source tools and machine-learning approaches that can model many aspects of proteomics data collection with remarkable accuracy. There are growing concerns about data privacy, patient consent, and potential personal identifiability (see General Data Protection Regulation (GDPR) in Europe or the US Health Information Portability and Accountability Act).

This has caused many investigators to decline the deposition of human plasma and serum data sets in public repositories. To mitigate this problem somewhat and continue to foster some degree of availability of such valuable data, there are efforts underway at PRIDE^65^ and MassIVE^66^ to develop controlled access data repositories, wherein data sets with public-access concerns may be deposited with gated access in the hope that the data sets can still be used with proper justification and safeguards. In addition, many high-quality data produced for or by non-academic institutions have not been shared with the community, limiting the community’s possibilities to learn more about proteins in circulation.

Apart from the MS-based proteomics, community efforts around the PEA platforms have been started with the SCALLOP (http://www.scallop-consortium.com) and other consortia using this assay. These consortia are not public repositories, but new members are welcome to share data and benefit from shared data. Current data repositories are often cohort-centric, and public portals are being modified to host affinity proteomics alongside other omics data types. Thus, there will likely be many studies, like the UK Biobank,^22,68,69^ where the community can obtain affinity proteomics data.

Coordinated access to affinity data would greatly benefit the research community, as demonstrated by reanalyzing MS-based proteomics data available through public repositories. A first guideline for submitting affinity proteomics data to PRIDE (PRIDE-AP) has been suggested.^70^ This is particularly valuable for discovery-driven research, where there is still a limited content overlap among different platforms, which will change with technological advancements. For instance, of the 4608 proteins listed in the 2023 Human Plasma Proteome PeptideAtlas, 44% are covered by the 5416 proteins of the Olink Explore HT and 74% by the 9655 proteins of the SomaScan 11k Assay.^71,72^

## 2023 Build of the Human Plasma Proteome PeptideAtlas

The Plasma PeptideAtlas has been providing a periodic snapshot^2,3,73,74^ of the human plasma and serum proteomes based on public data-dependent acquisition (DDA) MS data sets since the first plasma build in 2005^76^ as part of the HUPO Plasma Proteome Project (HPPP).^4^ Fifteen years ago, data sets had to be solicited directly from producers since public repositories were in their infancy. Now, data sets are downloadable from public data repositories such as ProteomeXchange,^62^ converted to mzML, and processed through a uniform pipeline of sequence database searching and postsearch validation with the Trans-Proteomic Pipeline.^77^ Of note, there are around 125 queries per week for the Plasma PeptideAtlas build.

The 2023-04 build (serum and plasma combined) comprises 113 data sets, a 63% increase over the previous 2021-7 build, and yielded 108 million peptide-spectrum matches (PSMs), an 83% increase since the previous build. The number of distinct peptides detected increased by 27% to 0.26 million, whereas the total number of canonical proteins increased by a mere 213 to 4608. Canonical proteins require at least two non-nested uniquely mapping peptides (≥9 amino acids) that together extend at least 18 amino acids, as described in the HUPO Human Proteome Project (HPP) mass spectrometry data interpretation guidelines.^78^ These 4608 proteins mapped to the core set of 19 778 PE1-4 core human protein entries in the neXtProt^79^ resource represent 23% of the human proteome, with various isoforms and immunoglobulin variation not counted in these estimates. At the stringency of the PeptideAtlas build, only a single decoy protein achieved canonical status, yielding an estimated false discovery rate of <0.04%. All proteins, peptides, and individual spectra supporting the identifications may be browsed at the PeptideAtlas website beginning at the top page for this build at https://peptideatlas.org/builds/human/plasma/.

**Figure 3:**
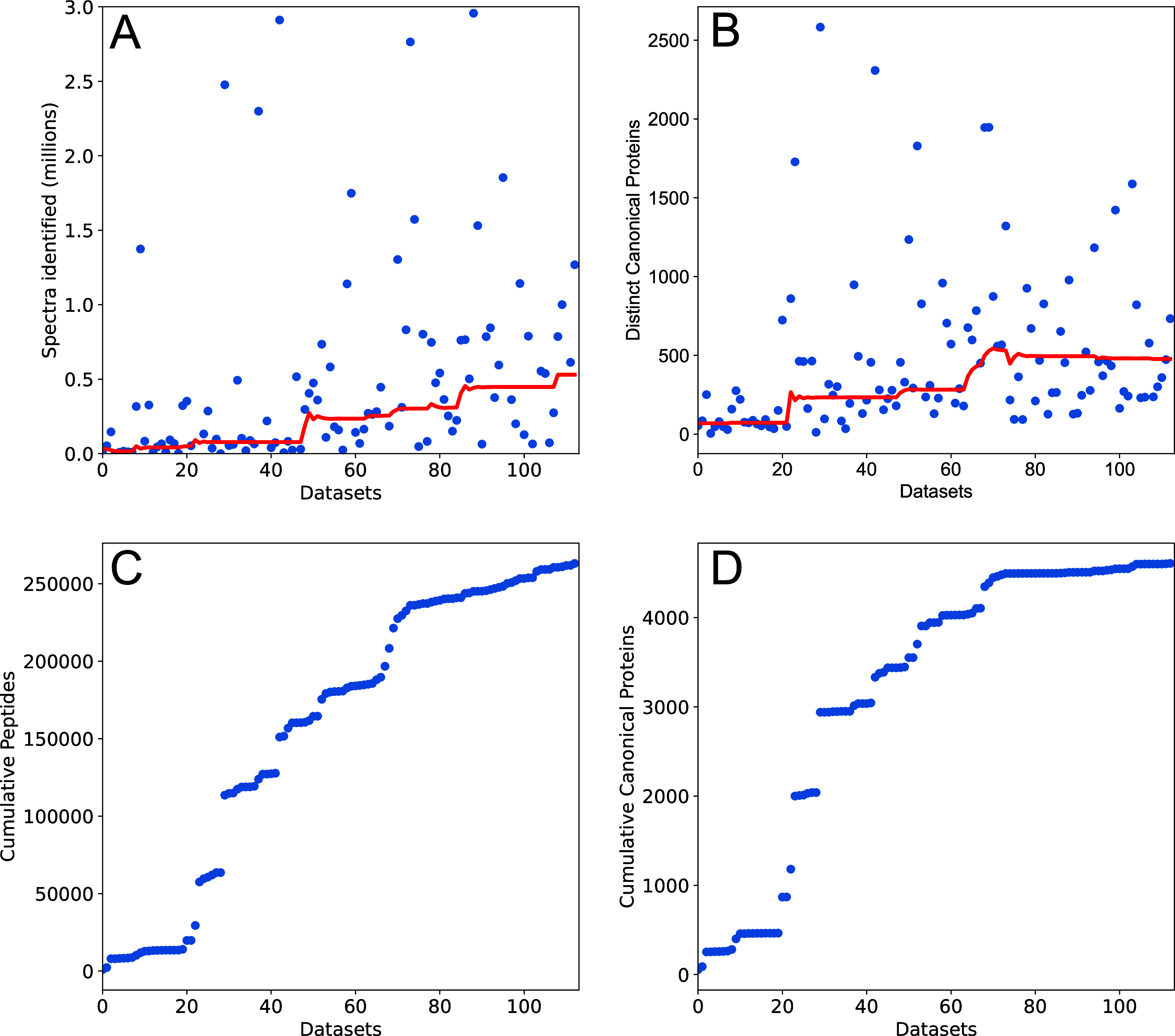
Trends from the 2023-04 build of the Human Plasma PeptideAtlas. (A) Number of identified spectra over time as more data sets are added. The axis is truncated to omit data sets with very high spectrum counts. A median-filter trend indicates an increase in the typical number of identified spectra per data set from 50 000 15 years ago to half a million spectra today. (B) The number of distinct canonical proteins identified over time as more data sets are added to the PeptideAtlas build. The typical number of canonical proteins detected has risen from 100 to 200 in early data sets to 500 proteins today in DDA. (C) The number of cumulative peptides in the Plasma PeptideAtlas continues to increase. The top axis provides the approximate years when data sets were added to the Plasma PeptideAtlas. (D) The number of proteins added per data set has slowed substantially, increasing by only 200 proteins despite the recent addition of nearly 50 million PSMs.

Figure 3 provides a view of trends over the lifetime of the Plasma PeptideAtlas including trends in the number of spectra identified per data set and the number of distinct canonical proteins per data set. Although modern data sets generate substantially more spectra that can be identified, the overall trend in the number of identified proteins is modest. The range is still quite large, driven primarily by the amount of fractionation performed in a study. Here, technological developments are needed to enable in-depth high-throughput analysis.

The rapid technological advancements in terms of higher proteome coverage in MS-based proteomics, especially facilitated by the combination of enrichment and precipitation methods and the novel Astral mass spectrometer, will continue to enrich the number of identifications. This will likely also be reflected in future builds of the PeptideAtlas.

**Figure 4:**
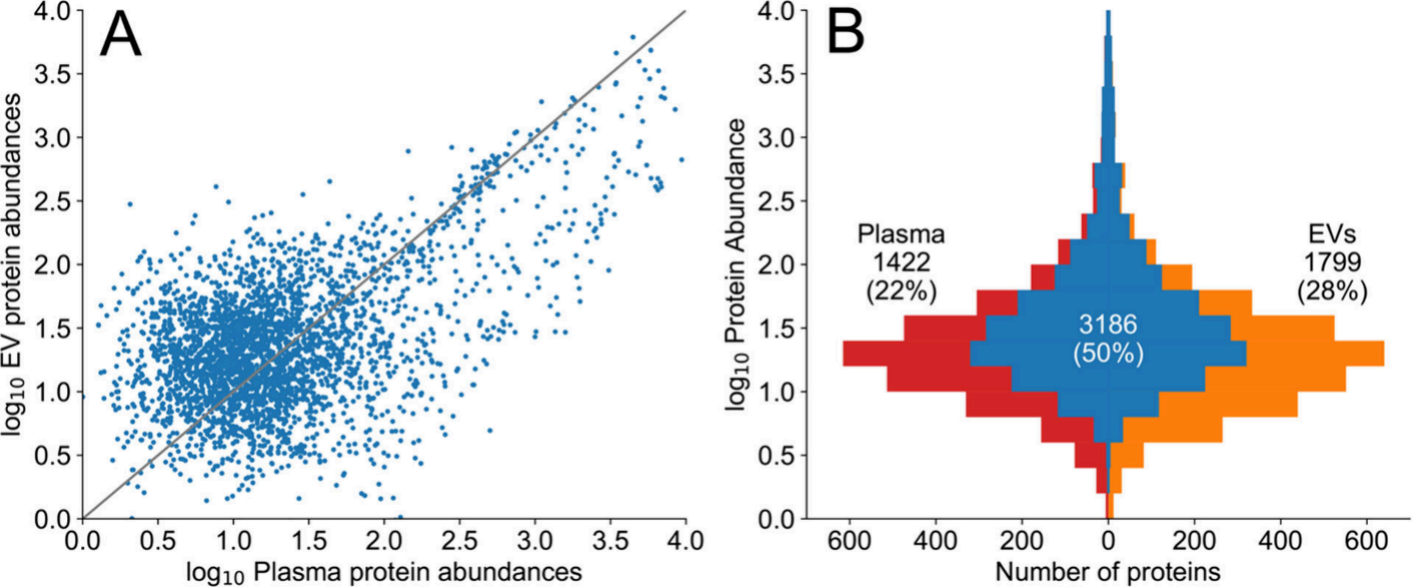
Comparison of protein abundances in plasma/serum samples versus EV samples. (A) Protein abundance estimated from log_10_ PSM counts for proteins seen in both PeptideAtlas builds. Most proteins correlate between plasma/serum quite well. Still, a noticeable population of proteins has a much higher abundance in plasma/serum than in EVs, while the opposite is not observed. (B) Overlap in protein identifications (blue) between plasma/serum build (red) versus EV build (orange), displayed as a function of estimated abundance (log_10_ PSM counts). 50% of proteins are seen in both builds, with ∼25% unique to each build.

In 2021, PeptideAtlas introduced a build comprising only data from extracellular vesicles extracted from blood.^2^ The 2023-04 build of the Human Extracellular Vesicle PeptideAtlas expands on this to include 33 PXDs yielding 10 million PSMs, 181 045 distinct peptides covering 4985 canonical proteins, just 377 more canonical proteins than the plasma build. Relative abundances of plasma and EV proteins are crudely estimated based on the number of PSMs that map to each protein and are compared to each other in Figure 4A. With a similar number of proteins overall in the two builds (although a factor of 10 more PSMs in the plasma build), general abundances correlate well for many proteins. Figure 4B shows the overlap in protein identifications in the two builds as a function of abundance, illustrating the overabundance of classical plasma proteins in EV data. A very high overall overlap is evident in the upper 2 orders of magnitude, but only ∼50% overlap is observed in the lower 2 orders of magnitude.

## Post-translational Modifications—Beyond the Canonical Proteome

In addition to canonical proteins, the landscape of post-translational modifications (PTMs) offers a biologically relevant layer to characterize the circulating proteome. PTMs affect proteins in many ways, including solubility, structure, ligand binding, localization, stability, secretion, and activity. With more than 400 known modifications, LC-MS is a powerful technology to study a plethora of PTMs, such as glycosylation, phosphorylation, methylation/acylation, and ubiquitination.^80^ An additional layer of complexity arises from the crosstalk between PTMs, leading to intricate combinations of modifications fine-tuning biological outcomes. Historically, most investigations only focused on one type of PTM, but more recently, PTM combinations and crosstalk have been characterized and studied.^81,82^ Here, we highlight two types of PTMs that are found in the plasma proteome: glycosylation and phosphorylation.

While it is well-known that changes in glycosylation are associated with normal and disease-related processes, analytically characterizing those changes, including both the glycan/glycoprotein identity and their abundance, can be challenging.^83^ Recent advances to address some of these challenges include (i) the development of chemical or biochemical tools to enrich glycoproteins or glycopeptides from complex mixtures, (ii) methods to resolve the site and families of glycosylations, (iii) approaches for targeted glycoproteomic analysis, and (iv) advancements in bioinformatics to improve the confidence in glycan/glycopeptide assignments.^84−87^ The increased interest in glycoproteomics has resulted in some impactful observations, particularly for biomarker discovery as outlined below.

Many cancer biomarkers used in the clinic are glycoproteins, including carcinoembryonic antigen (CEA), CA125 (the repeating peptide epitope of the mucin MUC16), prostate-specific antigen (PSA), and alpha-fetoprotein (AFP).^88−90^ As MS-based methods for glycan characterization evolved, extensive characterization of these glycoproteins revealed highly heterogeneous structures, sometimes with 10s to 100s of different glycoforms present, such as with CEA and PSA.^91,92^ In a recent review, Chernykh et al. highlighted how advancements in LC-MS have enabled a new era of structurally resolved glycoproteomics, where researchers can now routinely assign thousands of N-linked and/or O-linked glycan structures.^93^ Such methods have made it possible to explore the correlation in expression levels between parent proteins and their glycoforms, leading to the correlation of specific glycoforms and phenotypes. In a proof-of-concept study investigating genetically defined glycosylation diseases, subtle (but known) changes in site-specific N-linked glycans have been used to stratify patients according to their specific genetic defects,^94^ highlighting the biological significance of glycoproteomics. Multiple-reaction monitoring (MRM) was used to assess the abundance of 159 predefined glycopeptides in healthy human plasma, while it has also been utilized to map intra- and interprotein glycan correlations. While in this study such correlations were used to stratify age– and gender–glycan relationships, the same principles could be applied to biomarkers for disease detection. Interestingly, the ability to characterize changes in specific glycoform levels in circulation likely results from changes in glycan-processing enzymes’ expression, which are often dysregulated during disease progression.^90^

Another emerging opportunity for applying glycoproteomics is improving predictions for functionally relevant and disease-subtyping efforts. Recent studies in ovarian, prostate, kidney, pancreatic, and liver cancers illustrate the contribution of glycoproteins to the prediction of clinical outcomes, the severity of disease progression or risk, identifying specific pathological subtypes, or monitoring treatment pathways.^95−99^ Although most current efforts utilize tissue as their primary source material, we wanted to highlight opportunities to build off this foundation and extend the results into the circulating proteome. Of note, early-stage diagnostic companies are utilizing these glycosylation changes to build blood-based diagnostic tests for early disease detection.

Like protein glycosylation, phosphorylation has also been observed in the blood proteome. While there are many mechanisms under which these modified proteins appear in circulation, three mechanisms include Golgi-mediated phosphorylation to actively secreted proteins, secreted kinases that modify proteins extracellularly, and leakage proteins from cell death or related events.^100,101^ Several large-scale discovery studies have helped catalog some of these extracellular and circulating phosphoproteins, revealing interesting mechanisms related to disease progression, treatment, and/or monitoring.^102,103^

A major proteogenomics project is the National Cancer Institute-sponsored Clinical Proteomics Tumor Analysis Consortium (CPTAC), now in its 17th year. Multiomics analyses of drivers and targets for translation to oncology have been published for a broad array of cancers. For example, clear cell Renal Cell Adenocarcinomas account for 75% of kidney cancers. Clark et al.^104^ identified remarkable features of 3p loss, translocations, and biallelic mutations of VHL (nearly universal) in combination with mutations of one of four tumor suppressors coded on chromosome 3p (PBRM1, BAP1, SETD2, KDM5C). Phosphoproteomic analyses showed transcription factors specifically involved in PI3K-mTOR or HIF-2alpha regulatory pathways, cell cycle, or angiogenesis. Many findings with proteomics or phosphoproteomics could not be predicted from DNA or RNA analyses. Kinase targets for many chemotherapy agents approved by the FDA or in clinical trials were identified. Four subtypes of ccRCC were identified that could guide use of immunotherapy regimens: CD8+ inflamed, CD8– inflamed, metabolic immune desert, and VEGF immune desert. Further work reported features associated with aggressiveness/poor survival of ccRCCs, including UCHL1 as a biomarker of survival, and characterized rare types of kidney tumors, including papillary, chromophobe, angiomyosarcoma, and oncocytomas.^98^

Similar studies have been published on lung adenocarcinomas (LUAD), pancreatic ductal adenocarcinomas (PDACs), and many other cancers.^105−108^ An ancillary study identified circulating serum biomarkers presumably released from PDACs into the circulation.^105^

## Networks and Connections of Circulating Proteins

One approach to defining subproteomes is their coregulation in groups or networks, which might pinpoint distinct regulatory networks. The reason for coregulation can be found in various mechanisms, from organ or tissue leakage to the presence of circulating cells, stable protein complexes, aggregation to lipoprotein particles, EVs, or the response to specific stimuli.

Typically, tissue leakage proteins are clinical markers of organ damage (see troponin) or are used to detect cancers due to the aberrant secretion of cancer-associated proteins, such as CA125, for early detection of ovarian cancer. Next to the disease context, tissue leakage proteins can originate from biological processes. However, due to their relatively low abundance, tissue specificity, and less consistent presence, detecting a wider variety of tissue leakage proteins has been very limited to severe phenotypes such as COVID-19.^109^ Using isoelectric focusing of peptides and MS-based readout, a recent study reported placenta-specific proteins released in circulation during pregnancy.^110^ Combining this with genetic information (denoted *proteogenomics*) has allowed the study to assign those detected peptides with specific single amino acid variants to either the fetus or the mother. This demonstrated a cross-placenta exchange between the child and the maternal circulation system. In a study of alcohol-related liver disease, paired liver–plasma samples could be used to discern tissue damage markers and proteins involved in active signaling processes.^111^

In addition to applying differential abundance statistics to identify coregulated proteins, network-based approaches are a valuable tool to gain a deeper biological understanding of proteins or reduce collinearity for downstream statistical analysis. This is especially true for proteins with low-fold changes or high interindividual variation, which is the case for a large proportion of the proteome.^6,112,113^ Coexpression network approaches, such as weighted (gene) coexpression network analysis (WGCNA), use pairwise association measures to describe the correlation relationships between analytes.^113−115^ The information content of pairwise correlation analysis can be very extensive. For example, a study analyzing 1000 proteins would generate a matrix of 499 500 correlations. When combining the correlation matrix with a clustering algorithm, “global correlation maps” can be constructed, and organizational features pointing to physical connections or functional associations can be captured.^116^ While correlation does not prove causation, associations with clinical parameters or anthropometric information can generate hypotheses for underlying biological mechanisms. Combining these with enrichment analysis using GO or KEGG terms can reveal further biological clues about the origin of the observed correlation cluster. Setting the limit to the included proteins, such as for the targeted affinity-based assays, or the currently technically possible markers, such as for MS-based discoveries, could reveal different findings than including the whole proteome. Another key to facilitating a more informed outcome is the sample size, with more extensive studies increasing the power to detect small effect sizes, complex signatures (i.e., many proteins comprising a unique pattern), or changes associated with cohort subsets. For example, studying serum samples of 5000 Icelanders identified regulatory networks of circulating proteins that pointed at coordinated regulation modules across human tissues associated with health outcomes and genetic variants linked to complex diseases.^117^ A study analyzing 2622 samples demonstrated how to connect protein coexpression modules with clinical variables to interpret the underlying mechanisms of coexpressed proteins.^118^ Another study drafted an extensive list of associations between proteins and clinical parameters in COVID-19, providing a treasure trove to interpret protein coexpression networks.^119^ This can be further grown to interomic cross-sectional correlation networks, including proteomics, clinical parameters, microbiome, genetic traits, and metabolomics,^120,121^ with some statistical tools such as multiomics factor analysis enabling differentiation of biological and technical factors.^122^

Technological advancements resulting in fewer missing values, higher throughput for larger studies, higher reproducibility, and tailored multiomics integration strategies will make network-centric analysis more robust, powered, and insightful. Increasing data amount and quality via higher proteome coverage will increase our knowledge about organ-centric and systemwide regulation of the circulating proteins.

## The Extracellular Vesicle Subproteome

Extracellular vesicles (EVs) comprise a heterogeneous group of lipid bilayer particles secreted by cells and play an important role in cell-to-cell communication and cargo transportation processes. There are three main subgroups of EVs, which are classified primarily according to their sizes and biogenesis: exosomes, microvesicles, and apoptotic bodies. Exosomes are small EVs with diameters ranging from 30 to 150 nm, which are generated inside the cell in multivesicular bodies (MVBs) and subsequently released through the fusion of the MVB with the plasma membrane. Microvesicles are larger, 100–800 nm in diameter particles shed directly from the cell’s plasma membrane, similar to apoptotic bodies (200 nm–5 μm) created specifically during apoptosis. EVs carry nucleic acids, such as microRNA and mRNA, lipids, and proteins, which can be transferred to the recipient cell. Studies have shown that the content of EVs differs depending on their cellular lineage and that they reflect the cells from which they originate. In addition, they offer a more accessible, cell-like dynamic range of protein concentrations. These factors make EVs highly interesting as a potential source of biomarkers and vehicles in drug delivery. From a circulating proteome perspective, EVs provide a shielded, partially hydrophobic, and nonoxidative entry mechanism into plasma. They may contain proteins (e.g., strictly intracellular proteins) not commonly detectable in circulation. They are large enough to be retained in circulation during kidney filtration. They are an integrated part of the circulating plasma proteome, albeit at relatively low concentrations (∼10^10^ particles/mL). Studying the population of EVs in plasma (pEVs) is associated with some distinct challenges, mainly due to the heterogeneity of the particles, their relatively low abundance in plasma, and their physicochemical similarity to highly abundant, non-EV complexes in plasma, such as LDL and chylomicrons, leading to coenrichment during EV isolation. This has hampered the analysis of pEVs. Moreover, the number of identified proteins from MS analysis of pEVs is generally substantially lower than from EVs isolated from cell culture. Several enrichment techniques have been developed to enrich EVs from plasma, the most common strategies being based on ultracentrifugation, size exclusion, and immunocapture of combinations thereof. These techniques have individual advantages, but all suffer from unique challenges, including specificity, scalability, and reproducibility. Recently introduced strong anion exchange (SAX) magnetic beads have been applied for binding to phospholipid bilayer-bound particles.^123^ The direct combination of protein aggregation capture (PAC) sample preparation with the magnetic particles has resulted in an automated workflow seamlessly integrating enrichment and proteomics sample preparation. Several studies have reported markers for the different EV subgroups, including tetraspanins and Alix for exosomes, actinin-4, and HSP90B1 for microvesicles. However, individual EVs are very heterogeneous, providing a challenge for selecting immunocapture targets and distinguishing between a true EV protein and a coenriched protein. Notably, the International Society of Extracellular Vesicles (ISEV) has published the “Minimal Information for Studies of Extracellular Vesicles” (MISEV https://www.isev.org/misev) guidelines since 2014. This effort should guide the standardization of protocols and reporting in the extracellular vesicle field to improve the reproducibility and quality of the EV studies. This is crucial because minor changes in protocols, such as choosing a consistent sample starting material (plasma preparations or serum), can have a significant effect on the population of EVs.

In recent years, a substantial amount of research has been put into defining subpopulations of EVs based on the presence of different surface markers, often using combinations of immunocapture and global MS analysis.^124−127^ These studies provide an in-depth characterization of the diverse EVs found in plasma, identifying 1000–2000 proteins, but often at the cost of low throughput and high starting volume. These studies clearly show the potential of detecting EVs from defined cell populations, such as platelets^125,128^ or specific tissue-derived EVs.^126^

## (Auto)antibody Isotyping

Antibodies are a key constituent of the circulating proteome, an essential part of the immune system, key players in defending against pathogens, and invaluable tools in diagnostic and therapeutic applications. Therefore, comprehensive profiling of the antibody repertoires provides crucial insights into immune responses and underlying disease mechanisms. Isotyping of antibodies can be challenging due to multiple isotypes, cross-reactivity among isotypes, and the low abundance of antibodies in some instances, making their detection and characterization arduous. Additionally, the dynamic nature of autoantibody profiles and the potential for isotype switching further complicate antibody isotyping results’ interpretation and clinical relevance. Mass spectrometry is emerging to provide added information on sequence diversity and dynamics of complex antibody mixtures—information that cannot be obtained through classical immunoassays or traditional sequencing of B-cell receptor repertoires at the nucleotide level.^129^

A recent study used personalized IgA1 Fab clonal profiles from human serum and milk samples.^130^ In line with previous findings,^131−133^ each donor exhibits a unique repertoire of clones, and each clonal profile was considerably stable across longitudinally collected samples. The authors found that serum and milk IgA1 are dominated by a few clones, with a significant overlap arising from dimeric J-chain coupled IgA1. Conversely, the clonal repertoire of monomeric serum IgA1 was less shared, indicating the existence of two distinct sources (B cells) for the two IgA1 repertoires.^130^

MS-based antibody profiling has also shed light on the polyclonal response of individuals producing SARS-CoV-2 S-protein-targeting IgG1 clones,^134^ greatly supporting serological assays,^135−138^ which lack the resolution to discern the unique antibody clones. Additionally, mapping post-translational modifications (PTMs) dramatically improves our understanding of the antibody’s efficacy and stability, as shown recently by linking IgG fucosylation to the severity of COVID-19^139^ and secondary Dengue infection.^140^

Understanding the specific roles of antibodies will remain of high interest. For example, binding to endogenous proteins is crucial in diagnosing and treating autoimmune diseases. In the latest systematic efforts, text-mining, statistical analysis, and manual curation were used to develop a web interface that currently presents >8000 human autoantigens (AAgs) as part of the AAgAtlas portal (http://biokb.ncpsb.org.cn/aagatlas_portal/index.php^141^) containing 8045 nonredundant AAgs and 47 post-translationally modified AAgs against 1073 human diseases. Predominantly using immunoassay data, immunogenic properties of human AAgs were classified based on their genetic, biophysical, cytological, expression profile, and evolutionary characteristics. These data outline some of the hallmarks of human autoimmunity, hence presenting the value of using blood samples for the deeper characterization of the antibody repertoires and clonalities across human diseases.

## Multiplexed Serology

Compared to autoantibodies, antibodies are produced in response to pathogen infections. Proteomics technologies have been developed to survey the serological profiles of infections. To exemplify their utility, a SARS-CoV-2 proteome microarray containing 966 tiled 15-mer peptides was developed to profile the B-cell epitope landscape of SARS-CoV-2 IgM and IgG antibodies in early COVID-19 infection.^142^ Others constructed a SARS-CoV-2 protein array containing 18 purified viral proteins to detect the IgG and IgM antibody responses.^143^ In addition, antibody profiles of COVID-19 patients and prepandemic controls were analyzed using immunoprecipitation and sequencing (PhIP-Seq). Here, an oligonucleotide library encoding 56-mer peptides across the proteomes of all known pathogenic human viruses (∼400 species and strains) identified more than 800 highly selective epitopes in the SARS-CoV-2 proteome by machine learning.^144^ Compared to these proteomewide analyses, high-sample-throughput serology assays using suspension bead arrays have been applied to home-sampled dried blood spots (DBS).^145^ Altogether, these antibody-centric studies of the circulating proteome illustrated the systemic view of the immune response against human and pathogenic targets.

## Microsampling Opportunities

Blood-sampling techniques and preparatory processes have evolved to meet the requirements of individual clinical tests but have primarily not been optimized for use outside clinical settings. For cost-effective, offsite monitoring of subjects, the ideal sample should be collected quickly and robustly, with minimal involvement of clinical personnel, and easily stored while containing a wide array of biomolecules responsive to health and disease states. Recently, microsampling has reemerged to collect minimal invasive sample volumes, such as blood drops or interstitial fluid. Such samples can contain valuable biological information from proteins, metabolites, and lipids.^146−149^

These approaches offer advantages, including not requiring a phlebotomist or clinician and opening new avenues for understanding disease phenotypes through repeated longitudinal sampling. Patient-centric sampling methods can facilitate population-scale health assessments and high-frequency sampling to capture time-resolved changes in molecular phenotypes, such as during exercise or drug trials. However, the robustness of this sampling method across larger cohorts and the resulting analytical insights remain to be explored and confirmed.

Volumetric microsampling devices have emerged as alternatives to traditional dried blood spot (DBS) sampling, primarily due to their capability to collect precise and consistent sample volumes (e.g., 10, 20, or 30 μL). These devices also address the inherent challenge of volumetric hematocrit effects associated with DBS sampling. Alternative setups that filter blood cells recently emerged but have not been tested and proven to improve the analytical performance compared to classical whole blood collections. Their user-friendly nature, precision, and convenient storage make them a popular and cost-efficient alternative in population-scaled research and clinical studies.^145,150^

However, there are limitations when applying deep proteomics analysis to samples collected using these microsampling devices. Tools and techniques optimized for plasma proteomics may not yield the best results when applied to these samples. Alternatively, optimizations specific to microsampling samples may be required. For instance, blood contains large amounts of erythrocytes that further challenge the limited dynamic range by masking analytes from readout detectors. Drying proteins can produce stable salt crystals that remain difficult to resolve. Eluting protein from such matrices may require detergents or chemical treatments that affect the suitability of the samples for proteomics assays. Moreover, the increased sample heterogeneity comprising soluble proteins, vesicles, and cells requires adjustments in protein extraction protocols.

## Conclusion and Outlook

The analysis of circulating proteins has gained growing interest, and several commercial providers (Table 1) have lowered the threshold to access robust technologies beyond the proteomics community. Historically, serum and plasma proteomics workflows have emerged from academic laboratories within the proteomics community. Given the significant promise of robust, deep, and accessible plasma proteome profiling, commercial platforms have recently emerged focusing on MS and non-MS readouts.^151^ It remains to be seen how the established and more recent platforms will complement each other to improve our understanding of circulating proteins (and besides being faster, cheaper, more sensitive, of higher throughput, and requiring low sample volumes).

**Table 1:** Platforms for Multiplexed Analysis of Circulating Proteins (Excluding Antibodies)^a^

provider	platform specific characteristics	features	batch size	read-out system	source
Alamar Bioscience	antibody-based proximity assays	250+	80	NGS	https://www.alamarbio.com/(16)
Biognosys	depletion for ultradeep analysis	2700	NA	MS	https://www.biognosys.com/(152)
BioTechne	semiautomated microfluidic system (ELLA)	1–8	16–72	fluorescence	https://www.bio-techne.com/
Encodia	amino acid degradation	**	**	NGS	https://www.encodia.com/
Luminex***	bead-based immunoassays	1–384	96–384	flow cytometer	https://www.luminexcorp.com(153,154)
MesoScale	sandwich immunoassays	1–10	96–384	electrochemiluminescence	https://www.mesoscale.com/
Nautilus Bioscience	cycling of antibody binding	20000*	**	protein array	https://www.nautilus.bio/(155)
Olink	antibody-based proximity assays	24–5300	44–88	qPCR, NGS	https://www.olink.com(10)
Quanterix	bead or disc-based nanowell assays	1–10	88	chemiluminescence	https://www.quanterix.com/(156)
QuantumSi	amino acid degradation	**	**	own system	https://www.quantum-si.com/(157)
Resyn Biosciences	ultrafast and shallow	4000	96	MS	https://www.resynbio.com/(123)
Seer	nanoparticle enrichment	6000	40	MS	https://www.seer.bio/(158)
Siscapa	immuno-affinity capture of target peptides	200	96–384	MS	https://siscapa.com(180)
SomaLogic	aptamers with slow off-rates	11000	80	DNA array, NGS*	https://www.somalogic.com(11,159)

a Stars indicate whether information was recently announced (*), is currently unknown (**), or if several providers of other kits and assays are present or have been developed by users (***).

Studying the circulating proteome is still an ideal showcase for technology-driven biology. We have and will continue to learn about personalized health signatures and how these can change during our lives due to the development of diseases. We will better understand the (many) processes in which pleiotropic proteins are involved and which sample, method, or phenotype-related traits influence protein detection and analysis. We will likely have many computational tools available to capture quality features and reveal a diversity of underlying phenotypes (e.g., use of medication, liver metabolism, or kidney damage) rather than sticking to the silos of categories of being grouped as healthy or diseased. The ease of access to blood will also allow us to use new sampling schemes to determine systemwide molecular health trajectories, such as organ-specific aging,^160^ at a much higher frequency than currently possible with clinical sampling and the available large biobanks. The hits and misses of blood proteome studies will likely influence how we study other but connected body fluids such as CSF, urine, or saliva.

In the coming years, we can expect an even more noticeable proportion of the circulating proteins to be reliably measured, ideally by several different technology platforms. While the growing adaptation, depth, throughput, and speed will be a natural development and find its use in a growing research community with interests in different types of biomarker studies (Table 2), it remains to be proven how these technological advances can add to biology and translate into tests that aid to improve or even save lives. New tools will be needed to test observations from blood analyses in functional and systemwide studies, where non-modifiable factors (e.g., genetic variation) and perturbations (e.g., lifestyle, infectious agents, or medication) can be decoded to gain mechanistic insights into the human ‘omes.^181^ In addition, the description of the circulating proteome will become more complete as soon as we can reliably add additional layers of complemtary information on top of protein levels, determine the protein interaction landscape in blood, capture the diversity of protein modifications and structural elements, and detect the array of single-molecule variants.

**Table 2:** Examples of Recent Protein Biomarker Projects Using Different Proteomics Techniques across Disease Areas

year	title of study^reference^	description of biomarker(s)	disease areas
2017	Role of Exosomal Proteins in Cancer Diagnosis^161^	several for different cancers	cancer
2020	Multiomic Blood Correlates of Genetic Risk Identify Presymptomatic Disease Alterations^162^	766 detectable alterations in proteomic, metabolomic, and standard clinical laboratory measurements	multiple
2020	Proteome Profiling in Cerebrospinal Fluid Reveals Novel Biomarkers of Alzheimer’S Disease^163^	26 protein panel	neurological
2021	A Time-Resolved Proteomic and Prognostic Map of COVID-19^164^	different panels for different outcome predictions like altered coagulation and inflammation	infection
2021	Plasma Proteome Fingerprints Reveal Distinctiveness and Clinical Outcome of SARS-CoV-2 Infection^165^	ADM, IL-6, MCP-3, TRAIL-R2, and PD-L1 predictive for death	infection
2021	Lung Proteomic Biomarkers Associated with Chronic Obstructive Pulmonary Disease^166^	25 proteins associated with COPD	infection
2022	Noninvasive Proteomic Biomarkers for Alcohol-Related Liver Disease^111^	nine protein panel for fibrosis, 12 protein panel for steatosis	metabolism
2023	Next Generation Pan-Cancer Blood Proteome Profiling Using Proximity Extension Assay^167^	different proteins and panels for specific cancer types	cancer
2023	Targeted Plasma Proteomics Reveals Signatures Discriminating COVID-19 from Sepsis with Pneumonia^168^	TRIM21, PTN, and CASP8	infection
2023	Elevated Plasma Complement Factor H Related 5 Protein Is Associated with Venous Thromboembolism^20^	CFHR5	cardiovascular
2023	Associations of Plasma Proteomics with Type 2 Diabetes and Related Traits: Results from the Longitudinal KORA S4/F4/FF4 Study^169^	protein panels for subtypes of prevalent prediabetes	metabolism
2023	Mass Spectrometry-Based Autoimmune Profiling Reveals Predictive Autoantigens in Idiopathic Pulmonary Fibrosis^170^	immunoglobulin profiling in plasma reveals autoimmune signature	respiratory
2023	Proteomics Reveal Biomarkers for Diagnosis, Disease Activity and Long-Term Disability Outcomes in Multiple Sclerosis^171^	11 proteins in CSF, none in plasma	neurological
2022	Stratifin as a Novel Diagnostic Biomarker in Serum For Diffuse Alveolar Damage^172^	eight proteins from SOMA scan are validated	respiratory
2023	Proteome Profiling of Early Gestational Plasma Reveals Novel Biomarkers of Congenital Heart Disease^173^	nine proteins predict CHD with high accuracy	cardiovascular
2022	Circulating Proteomic Panels for Risk Stratification of Intracranial Aneurysm and Its Rupture^174^	two sets of biomarker combinations to accurately distinguish IA from healthy controls (accuracy: 87.50%) or classify IA rupture patients (accuracy: 91.67%)	cardiovascular
2015	Prediction of Colorectal Cancer Diagnosis Based on Circulating Plasma Proteins^175^	five proteins as predictive diagnostic signature	cancer
2020	Extracellular Vesicle and Particle Biomarkers Define Multiple Human Cancers^176^	defined a panel of tumor-type-specific EVP proteins in TEs and plasma, which can classify tumors of unknown primary origin	cancer
2023	Absolute Quantification of Pan-Cancer Plasma Proteomes Reveals Unique Signature in Multiple Myeloma^177^	potential biomarker panel of seven protein targets for the diagnosis of multiple myeloma patients	cancer
2023	High-Throughput Plasma Proteomics to Define the Precursor Multiple Myeloma Proteome and Identify Candidate High-Risk Disease Biomarkers of Progression^178^	identified proteins that significantly distinguished monoclonal gammopathy of undetermined significance (MGUS), smoldering multiple myeloma (SMM), and NDMM from healthy donors	cancer
2023	Plasma Proteomic Associations with Genetics and Health in the UK Biobank^69^	protein quantitative trait locus (pQTL) mapping of 2923 proteins that identifies 14 287 primary genetic associations, of which 81% are previously undescribed	multiple
2023	Large-Scale Plasma Proteomics Comparisons through Genetics and Disease Associations^22^	Olink Explore 3072 data generated by the UK Biobank Pharma Proteomics Project1 on plasma samples from more than 50 000 UK Biobank participants and compared the results with those of a SomaScan v4 study on plasma from 36 000 Icelandic people2, for 1514 of whom Olink data were also available	multiple
2023	Rare Variant Associations with Plasma Protein Levels in the UK Biobank^68^	variant-level exomewide association study identified 5433 rare genotype–protein associations, of which 81% were undetected in a previous genomewide association study of the same cohort	multiple
2024	High Throughput Plasma Proteomics and Risk of Heart Failure and Frailty in Late Life^179^	18 individual proteins with consistent associations with risk of incident heart failure in mid- and late life	heart failure and frailty

As technological progress continues and proteomics data become increasingly utilized by researchers outside the proteomics community, we must remain transparent and primarily report how many proteins we could measure in all samples (and not the sum of all detected). We need to be aware of the increasing request for platform-independent reliability in identifying circulating proteins, especially for the newer entries, those of the lowest abundance, and those prone to be influenced by molecular and analytical variance.

- Mass spectrometry and affinity-based assays have enhanced the utility of studying the circulating proteome.
- Persisting challenges emphasize the necessity to ensure data quality and reliability to disentangle biology.
- Undervaluing the importance of how confounding factors hamper the sample integrity can decrease the potential to reproduce findings.
- Future perspectives advocate for a broader approach considering extracellular vesicles, additional categories, and relationships between circulating proteins.
- Alternative sampling strategies can be implemented to comprehend the dynamic of human health and disease.

Pending issues include the following:

- Providing evidence for the presence of all 20 000 proteins in circulation.
- Detecting all circulating proteins in all samples studied.
- Quantifying the concentration of circulating proteins across the abundance ranges.
- Cataloging the PTM and interaction landscape of circulating proteins.
- Defining quality and integrity criteria for biological samples.
- Implementing practices for preanalytical, interindividual, and genetic factors.
- Identifying the sources of unknown biases and variation.
- Annotating the proteoforms and their regulation in health and disease.
- Implementing novel sampling techniques for deep proteome analysis.

## Data Availability

Data are available at https://peptideatlas.org/builds/human/plasma/

## Glossary

antibody depletion: this technology removes proteins with a high concentration typically from plasma samples, allowing the detection of lower concentrated proteins by MS-based analysis
assays: tests or methods used to detect proteins
(auto)antibody isotyping: identifying different types and classes of antibodies in the body, which are crucial components of the immune system; this analysis provides insights into immune responses and disease mechanisms
biological variation: nontechnical differences in protein measurements and other biological molecules among individuals
biomarker: a biological factor found in blood, tissues, or other body fluids that can indicate a normal or abnormal process, a condition, or a disease; biomarkers are used to diagnose diseases, monitor diseases and response to therapy, and predict disease outcomes
biomarker discovery: the process of finding molecules in biological samples that can indicate the presence of a disease or health condition
circulating proteome: the collection of all proteins found in the liquid part of blood; scientists study these proteins to understand health and disease
confounding factors: external parameters that can affect study results, which need to be considered
dynamic range: the range between the proteins with the highest and the lowest concentration
extracellular vesicles: lipid particles released by cells that carry proteins and other molecules
false discovery rate: the percentage of incorrect identifications caused by performing a larger number of tests; a lower false discovery rate indicates more accurate results
glycoproteomics: the technology to study glycosylated proteins or peptides
liquid handling: these are automated robotic systems used in laboratories to process and analyze biological samples, such as blood or plasma, with high precision
mass spectrometry (MS) and affinity-based approaches: advanced techniques used by scientists to measure and analyze proteins in blood samples
microsampling: collecting tiny amounts of blood for analysis
peptides: short chains of amino acids, the building blocks of proteins; peptides are a preparatory product of proteins and play a role in protein analysis
plasma: a type of cell-free blood preparation that uses additives to prevent coagulation from occurring
post-translational modifications: changes or modifications of the molecular composition of proteins after they are made, affecting their structure, functions, and interaction partners
preanalytics: processes required to collect and prepare samples for analysis, like blood collection, storage, or shipment
protein abundance: the amount of a specific protein present in a sample; scientists measure protein abundance to understand the concentration levels of different proteins in the blood
protein interaction: how proteins interact with each other in the body, affecting various biological processes; scientists study these interactions to understand the functions of proteins
proteomics: the study of all the proteins in a cell, tissue, or organism; in this context, it refers to understanding the proteins present in blood
proteoform: versions or modifications of proteins
sample contamination: unwanted substances enter the blood sample and affect the outcome of test results
sample preparation: methods and techniques used to prepare biological samples for analysis
serum: a type of cell-free blood preparation where coagulation occurs and clotting factors precipitate
targeted proteomics: this technique allows scientists to focus on specific and preselected proteins or peptides in a sample, helping to detect and quantify them accurately.
